# Transient Osteoporosis of the Hip: A Radiologist’s Perspective

**DOI:** 10.7759/cureus.29384

**Published:** 2022-09-20

**Authors:** Muhammad Mehraiz Khan, Misha Imtiaz, Faisal Ehsan Cheema, Niempa Bacani, Sadia Anwar, Usman Ismail

**Affiliations:** 1 Diagnostic Radiology, Institute of Nuclear Medicine and Oncology Lahore (INMOL), Lahore, PAK; 2 Internal Medicine, Naeem Surgical Hospital and Maternity Complex, Lahore, PAK; 3 Internal Medicine, Avalon University School of Medicine, Phoenix, USA; 4 Internal Medicine, Mayo Hospital, Lahore, PAK

**Keywords:** diagnosis, avascular necrosis (avn), bone marrow edema, magnetic resonance imaging (mri), transient osteoporosis of the hip (toh)

## Abstract

Transient osteoporosis of the hip (TOOH) is a rare disorder of unknown etiology without any antecedent history of trauma. There is a sudden onset of acute severe pain and temporary osteopenia in the joint involved with associated radiological findings of bone loss and marrow edema. Magnetic Resonance Imaging (MRI) is the gold standard imaging modality for diagnosis and disease monitoring. The major goal of this case presentation is to emphasize the necessity to add TOOH as an important differential of sudden hip pain and to review the literature on this entity.

## Introduction

Transient osteoporosis of the hip (TOOH) was originally identified in 1959 by Curtiss and Kincaid in three pregnant women who presented with abrupt severe unilateral or bilateral hip pain and thigh discomfort [1]. Middle-aged males and pregnant ladies in their third trimester are more prone to this condition. Clinically, the patients have an immediate onset of intense pain that is progressive. Condition deteriorates with heavy weight-bearing and strenuous exercise leading to functional restriction of the involved extremity [2]. It normally cures on its own, although it might be worsened by fracture or development to avascular necrosis (AVN).

Initial plain radiographs are usually unremarkable, but peri-articular osteogenic changes can be observed at 21 to 42 days after the initiation of signs and symptoms [3]. Magnetic resonance imaging is the preferred diagnostic method since it may detect bone marrow edema within 48 hours of the onset of clinical manifestations. With normal subchondral regions, affected areas in TOOH show low signal intensity on T1W (T1 weighted MRI) images and high signal intensity on T2W images, as well as short tau inversion recovery sequences [4]. Treatment is usually conservative including limited weight-bearing, physical therapy, and non-steroidal anti-inflammatory drugs (NSAIDs).

## Case presentation

A 46-years-old female presented to the radiology department with a one-month history of severe progressive pain in the right groin and right leg. The pain was exacerbated by physical activity. The patient denied any history of trauma or heavy exercise. She had no significant past medical or family history. She underwent appendicectomy 10 years back. On physical examination, she had limping gait and restricted right hip movement. Blood tests including inflammatory markers, serum uric acid, and serum rheumatoid factors were negative. 

**Figure 1:**
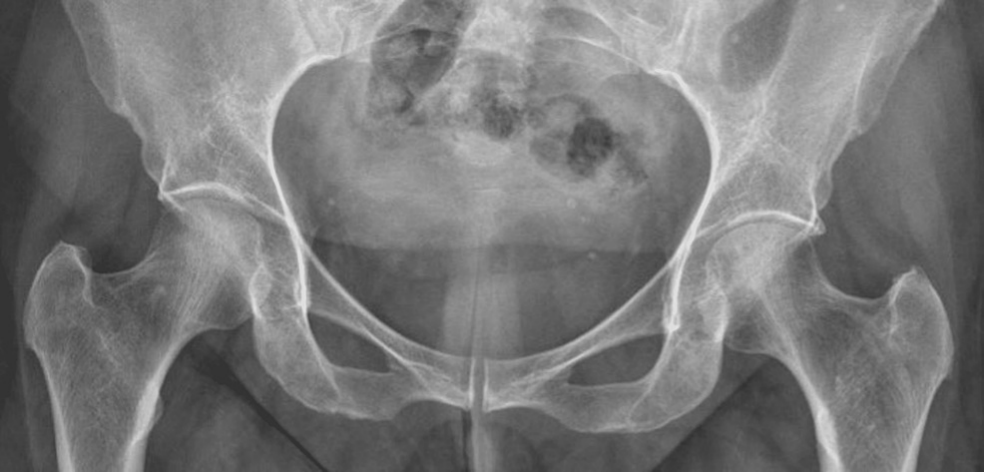
Plain radiograph of bilateral hip joints. The image shows intact joint spaces and no evidence of osteopenia.

**Figure 2:**
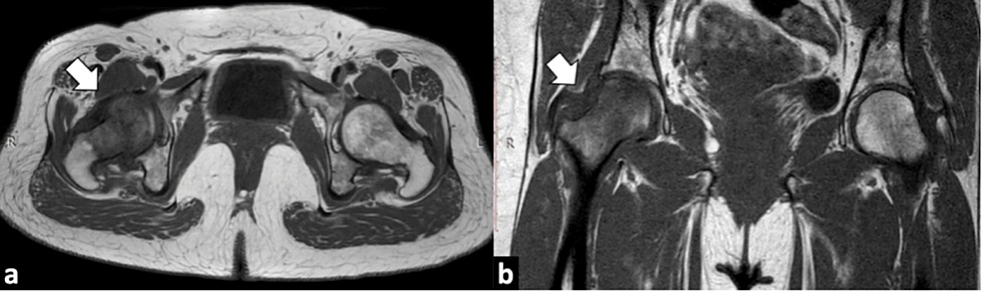
MRI hip images. Axial T1W image (a) and coronal T1W image (b) showing hypointense signals right femoral head and neck.

**Figure 3:**
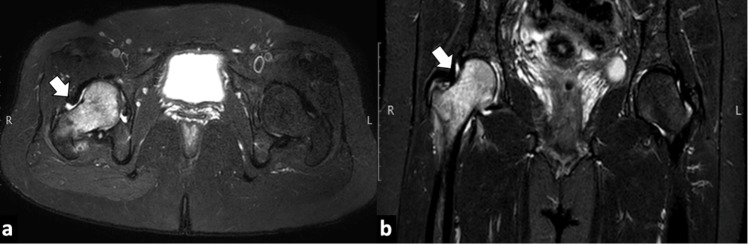
STIR imaging of the hip. Axial short TI inversion recovery  (STIR) (a) and coronal STIR images (b) show hyperintense signals in the head and neck region of the right femur due to marrow edema, findings are consistent with TOOH of the right hip joint.

A plain radiograph of the right hip was normal without any evident osteopenia, fracture, or joint effusion (Figure 1). MRI hip showed mildly reduced hip joint space on the right side. Abnormal signal intensity areas were noted in the head, neck, and trochanteric regions of the right femur appearing low on T1W (Figure 2) and high on T2W and STIR (short TI inversion recovery) sequences suggesting bone marrow edema (Figure 3).

**Figure 4:**
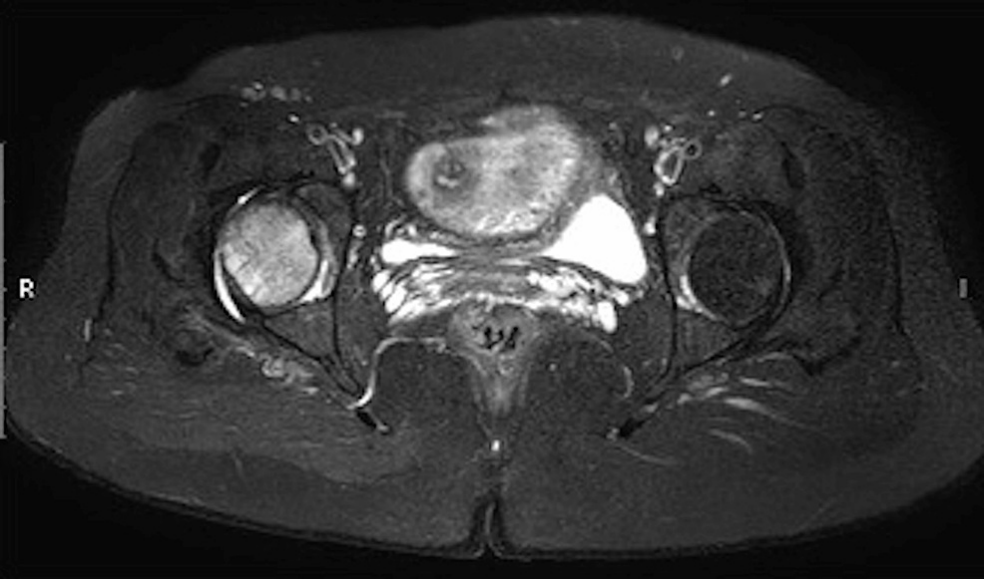
Axial STIR image of the pelvis at the level of hip joints. The STIR (short TI inversion recovery) image shows mild hip joint effusion on the right side.

**Figure 5:**
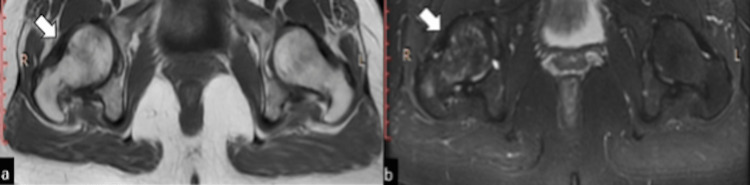
Follow-up STIR imaging at the level of hip joints Follow-up axial T1 (a) and axial STIR (b) images at the level of hip joints after 12 weeks show significant interval reduction in bone marrow edema of the right femoral head. Right hip joint effusion has also resolved. STIR: Short TI inversion recovery

Femoral head contours were preserved without evident features of osteonecrosis. Small right joint effusion was also appreciated (Figure 4). No established features of femoral head osteonecrosis or subchondral fracture were noted. On the basis of these imaging findings, a diagnosis of TOOH was made and the patient was sent back to her physician. The physician restricted her physical activity with non-weight bearing mobilization and gave calcium and vitamin D supplements. The patient’s symptoms started to resolve after three weeks and completely disappeared after 10 weeks. Follow-up MRI after 12 weeks showed a significant interval reduction in right hip marrow edema and joint effusion (Figure 5). Subsequent imaging findings confirmed the diagnosis of TOOH.

## Discussion

Transient hip osteoporosis is an infrequent cause of hip discomfort. It most commonly affects middle-aged males and pregnant ladies in their third trimester, with a male-female ratio of 3:1 [5]. Patients give a history of sudden onset of acute severe pain aggravated by physical activity and weight-bearing. The proposed mechanism of developing the condition is microtrauma, neurovascular and transient hyperemia leading to bone loss and marrow edema. Lower extremity bones or joints are the most commonly affected, the hip joint being the most common one, followed by the knee, foot, and ankle. Usually, one hip is involved at a time but a 20% incidence of bilateral disease was reported in a Japanese study [6]. Clinically, it manifests as a dull discomfort in the afflicted groin or buttock that radiates to the thigh and leg, along with functional impairment in the groin region. The pain worsens on bearing weight and progresses over weeks. Patients typically deny previous trauma or surgery.

Routine blood labs including hematological, bacteriological, and serological studies are usually normal. Although some individuals may have higher acute phase reactants, there are no definite biomarkers or other laboratory tests that aid in the diagnosis of TOOH. Elevated ESR and urine hydroxyproline levels are occasionally observed, indicating greater bone loss.

Plain radiographs may be normal or may reveal osteopenic changes. Because there is no radiographic evidence of demineralization in the early stages of the condition, advanced imaging is necessary. For quick identification of the disease and to begin therapy as soon as possible, magnetic resonance imaging (MRI) is the modality of choice. The appearance of bone marrow edema on MRI is a typical indication of TOOH. The edematous alterations of the bones are shown by an ill-defined region of hypointense signals on T1W imaging and hyperintense signals on T2W and STIR images. These radiographic abnormalities are frequently accompanied by associated joint effusion. Radioisotope bone scans have also been used to diagnose the condition, revealing higher radiotracer uptake in the affected areas [7].

There are two types of bone marrow edemas: reversible and irreversible. Causes of reversible edematous changes include transient osteoporosis, chronic regional pain syndrome, regional migratory osteoporosis, and trauma. Infection, avascular necrosis, malignancies, and degenerative and inflammatory arthropathies can cause irreversible edema. The self-limiting feature of transitory osteoporosis distinguishes it from other reversible causes of bone marrow edema [8]. For better patient care, it is critical to distinguish TOOH from other illnesses that have long-term consequences, such as AVN of the femoral head and destructive diseases of joints caused by inflammation and malignancy. On follow-up, patients of TOOH come with the resolution of symptoms clinically as well as on imaging.

## Conclusions

TOOH is a rare and self-limiting condition usually undergo undiagnosed. Its early diagnosis is crucial for better patient management and to avoid detrimental effects resulting from other diseases of similar presentation, which would otherwise leave the patient handicapped.
